# Down-regulation of cell surface CXCR4 by HIV-1

**DOI:** 10.1186/1743-422X-5-6

**Published:** 2008-01-11

**Authors:** Bongkun Choi, Paul J Gatti, Cesar D Fermin, Sandor Vigh, Allyson M Haislip, Robert F Garry

**Affiliations:** 1 Department of Microbiology and Immunology, Tulane University Health Sciences Center, New Orleans, LA 70112, USA; 2 College of Veterinary Medicine, Nursing & Allied Health (CVMNAH), Tuskegee University, Tuskegee, AL 36088, USA; 3 Department of Structural & Cellular Biology, Tulane University Health Sciences Center, New Orleans, LA 70112, USA; 4 Departments of Environmental Medicine, Pathology, and Medicine, New York University School of Medicine, Tuxedo, NY 10987, USA; 5 Biocompare, Inc., 395 Oyster Point Blvd, South San Francisco, CA 94080, USA

## Abstract

**Background:**

CXC chemokine receptor 4 (CXCR4), a member of the G-protein-coupled chemokine receptor family, can serve as a co-receptor along with CD4 for entry into the cell of T-cell tropic X4 human immunodeficiency virus type 1 (HIV-1) strains. Productive infection of T-lymphoblastoid cells by X4 HIV-1 markedly reduces cell-surface expression of CD4, but whether or not the co-receptor CXCR4 is down-regulated has not been conclusively determined.

**Results:**

Infection of human T-lymphoblastoid cell line RH9 with HIV-1 resulted in down-regulation of cell surface CXCR4 expression. Down-regulation of surface CXCR4 correlated temporally with the increase in HIV-1 protein expression. CXCR4 was concentrated in intracellular compartments in H9 cells after HIV-1 infection. Immunofluorescence microscopy studies showed that CXCR4 and HIV-1 glycoproteins were co-localized in HIV infected cells. Inducible expression of HIV-1 envelope glycoproteins also resulted in down-regulation of CXCR4 from the cell surface.

**Conclusion:**

These results indicated that cell surface CXCR4 was reduced in HIV-1 infected cells, whereas expression of another membrane antigen, CD3, was unaffected. CXCR4 down-regulation may be due to intracellular sequestering of HIV glycoprotein/CXCR4 complexes.

## Background

Chemokine receptors are seven-transmembrane G-protein-coupled receptors that upon ligand binding transmit signals, such as calcium flux, resulting in chemotactic responses [1-3]. Chemokine receptors are divided into four families that reflect differential binding of the CXC, CC, CX3C and XC subfamilies of chemokines [4]. Several members of the chemokine receptor family function as coreceptors with the primary receptor CD4 to allow entry of various strains of human immunodeficiency virus type 1 (HIV-1) into the cells [5-8]. T-cell-tropic X4 HIV-1 use CD4 and chemokine receptor CXCR4 for entry into target cells, whereas macrophage-tropic R5 HIV-1 use CD4 and chemokine receptor CCR5. Dual-tropic strains can use either CCR5 and CXCR4 as co-receptors. In addition, CCR3, CCR2, CXCR6 (Bonzo/STLR6) among other chemokine receptors can function as coreceptors and support infection by a more restricted subset of macrophage-tropic or dual-tropic HIV strains [9,5,12].

CXCL12 (stromal derived factor 1 α/β, SDF-1α/β) is the natural ligand for CXCR4, whereas CC chemokines, CCL3 (macrophage inflammatory factor 1α, MIP-1α/chemokine LD78α), CCL3-L1 (LD78β), CCL4 (MIP-1β), and CCL5 (RANTES), are ligands for CCR5 [13-16]. CXCL12, CCL3, CCL4 and CCL5 as well as other natural and synthetic chemokine receptor ligands are able to inhibit cell fusion and infection by various strains of HIV-1, dependent or independent of co-receptor usage [17-21]. These findings have encouraged the development of antiHIV therapeutics targeting chemokine receptors [22-25].

Productive infection of CD4+ cells with HIV-1 markedly reduces cell-surface expression of CD4, which follows a classic mechanism for retroviral interference [26,27]. Down-regulation of CD4 by HIV-1 has been attributed to the formation of intracellular complexes consisting of HIV-1 envelope glycoproteins and CD4 receptors [28], although other mechanisms may also be involved in a cell type dependent manner [29,30]. Chemokine receptors, including CCR5 and CXCR4, can be down-regulated after binding of their respective chemokine ligands by a mechanism involving endocytosis of the complex [31-33]. The envelope glycoproteins of HIV-1 competitively antagonize signaling by coreceptors CXCR4 and CCR5 [34,35]. Exogenously added recombinant soluble HIV-1 surface glycoprotein (SU, gp120) can be coprecipitated from the cell surface into a complex with CD4 and CXCR4, that may lead to the formation of a trimolecular complex between HIV SU, CD4 and CXCR4 [36,37]. However, prior studies have suggested that although CCR5 coreceptors are down-modulated during infection by R5 HIV-1, CXCR4 co-receptor is not down-regulated after productive X4 HIV-1 infection [38]. CXCR4 was shown to be selectively down-regulated from the cell surface by HIV-2/vcp in the context of CD4-independent infection [39] or from cells infected with CD4-independent HIV-1 isolate that enters directly via CXCR4 [40]. Furthermore, exogenous expression of the HIV-1 Nef protein reduced cell surface levels of CCR5 or CXCR4 [41,42]. Here, we examine whether or not productive infection by HIV-1 alters the cell surface expression of CXCR4. Our results indicate that CXCR4 is down-regulated from the surface of CD4+ T-lymphoblastoid cells infected by HIV-1 and that HIV-1 Env and CXCR4 are colocalized in infected cells.

## Results

### HIV-1 infection down-regulates surface expression of CXCR4 in RH9 cells

**Figure 1:**
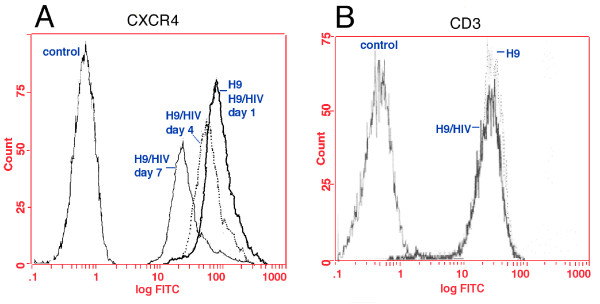
Flow cytometry analysis demonstrating reduced CXCR4 expression in HIV-1 infected RH9 cells. Panel A: RH9 T-lymphoblastoid cells infected with HIV-1_LA1_. On days 1, 4, and 7 postinfection cells were fixed with 4% paraformaldehyde, stained with mouse MAb 12G5 anti-CXCR4 (10 μg/ml) or isotype-matched control antibody followed by fluorescein isothiocyanate (FITC)-conjugated goat anti-mouse immunoglobulin G, and analyzed by flow cytometry. Median fluorescence intensity was calculated as an indicator of the level of cell surface CXCR4 expression. Data are presented as single-color histograms with FITC fluorescence (CD3 expression) along the horizontal axis and relative cell number along the vertical axis. RH9 cells (control cells), heavy solid line: H9 cells infected with HIV, dotted line; H9 with an isotype-matched control antibody, thin solid line. Panel B: Analysis of surface CD3 expression in HIV-1 and mock infected RH9 cells by FACS analyzed on day 7 post-infection.

To determine whether HIV infection alters cell surface CXCR4 levels, RH9 T-lymphoblastoid cells were infected with HIV-1_LA1 _at a MOI of 4 or mock-infected. At 1, 4 and 7 days post infection (PI), the level of cell surface CXCR4 on RH9 cells and HIV-1-infected RH9 cells were determined by flow cytometric analysis using CXCR4 monoclonal antibody (MAb) 12G5 [39]. Relative binding of 12G5 monoclonal antibody was significantly reduced compared to uninfected cells at 4, and 7 days postinfection, respectively (Fig. 1A). As a control, we also determined the effect of HIV infection on CD3 in RH9 cells. H9 cells infected with HIV maintained surface CD3 expression at a similar level to that of uninfected H9 cells (Fig. 1B). To determine the relationship between the expression of surface CXCR4 and HIV-1 protein expression, HIV-1 production by infected cells was quantified by a antigen-capture enzyme-linked immunosorbant assay (Ag-capture ELISA; Abbott Laboratories) and the number of HIV-1 antigen expressing cells were measured by indirect immunofluorescence microscopy. The decline in CXCR4 expression was accompanied by a rapid increase in HIV-1 protein expression in infected RH9 cells.

**Figure 2:**
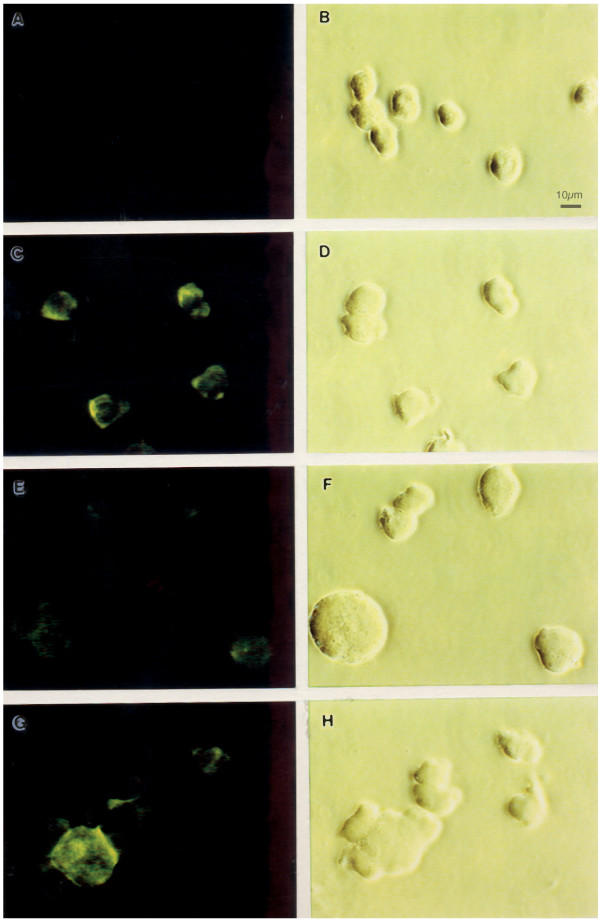
Immunofluorescence microscopy demonstrating reduced cell surface expression of CXCR4 in HIV-1 infected RH9 cells. Panel A: Immunofluorescence staining control with isotype-matched monoclonal antibody. Panel C: CXCR4 immunofluorescence staining of H9 cells. Panels E and G: CXCR4 immunofluorescence staining of H9 cells acutely infected by HIV-1. Panels B, D, F and H show phase contrast images of the same fields of cells shown in left panels. The fluorescent syncytial cell in panel G is representative of a minor population of cells in the infected culture (<10%) with a CXCR4 surface distribution similar to uninfected cells.

The results of these flow cytometric analyses were confirmed by immunofluorescence microscopy (Fig. 2). RH9 T-lymphoblastoid cells were infected with HIV-1_LA1 _at a MOI of 4 or mock-infected. At 4 days after infection cells were labeled with the CXCR4 12G5 MAb, followed by a FITC-conjugated secondary antibody and analyzed by indirect immunofluorescence microscopy. Whereas isotype-matched control antibody showed no reactivity (Fig. 2A, B), all control cells expressed CXCR4. The CXCR4-specific MAb displayed cell surface membrane fluorescence in 100% of mock-infected cells (Fig. 2C, D). Most cells in the HIV-1-infected cultures (>90%) showed markedly decreased surface CXCR4 staining (Fig. 2E–H), reflective of the flow cytometry results. The distribution of CXCR4 on the minor population of cells (<10%) with surface CXCR was similar to that of uninfected cells (Fig. 2G, H). HIV infection had no significant effect on the cell surface expression of CD3 indicating that decreased expression of CXCR4 is not a non-specific consequence of HIV-1 infection (not shown).

### HIV-1 infection induces internalization of CXCR4 in RH9 cells

**Figure 3:**
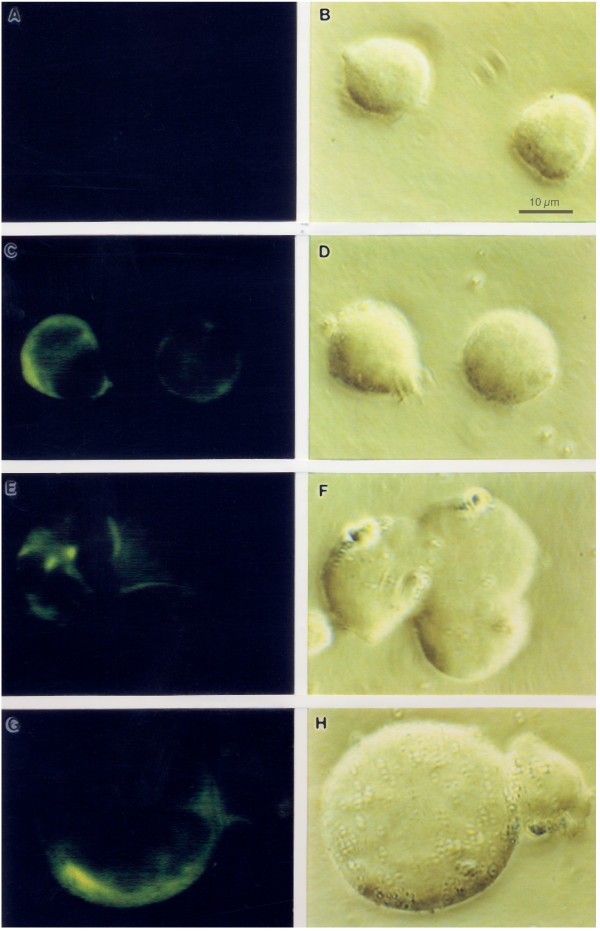
Immunofluorescence microscopy analysis of CXCR4 expression in permeabilized HIV-1 and mock infected RH9 cells. Four days after HIV-1 infection, cells were fixed, permeabilized with saponin and labeled with a mouse monoclonal antibody to CXCR4 (12G5) and a secondary, FITC-conjugated anti-mouse antibodies for observation with a fluorescence microscopy. Panel A: Immunofluorescence staining control with isotype-matched monoclonal antibody. Panel C: CXCR4 immunofluorescence staining of H9 cells. Panels E and G, CXCR4 immunofluorescence staining of HIV-1 infected H9 cells. Panels B, D, F and H show phase contrast images of the same fields of cells shown in left panels.

Down-regulation of surface CD4 by envelope glycoproteins from the plasma membrane has been attributed at least in part to the formation of intracellular complexes consisting of HIV-1 envelope molecules and CD4 receptors [26,43,44]. The potential internalization of CXCR4 in permeabilized HIV-infected H9 cells was investigated by immunofluorescence microscopy. RH9 T-lymphoblastoid cells were infected with HIV-1_LA1 _at a MOI of 4 or mock-infected. After 4 days PI, the cells were fixed, permeabilized by incubation with 0.05% saponin in PBS for 15 min to allow the entry of antibody and incubated with CXCR4 MAb followed by a FITC-conjugated second antibody. No fluorescence was observed in cells incubated with control antibodies (Fig. 3A, B). CXCR4-specific MAb 12G5 stained the surface of uninfected control cells (Fig. 3C, D). A weak additional intracellular signal observed in some control cells may be attributed to newly synthesized CXCR4 molecules in intracellular compartments of secretory pathways. In cultures productively infected with HIV-1, intracellular CXCR4 staining was markedly increased in approximately 50% of the cells, with a redistribution of the staining that is consistent with the intracellular accumulation of the receptor (Fig. 3E–H).

### HIV-1 SU and CXCR4 are colocalized in HIV-1 productively-infected RH9 cells

**Figure 4:**
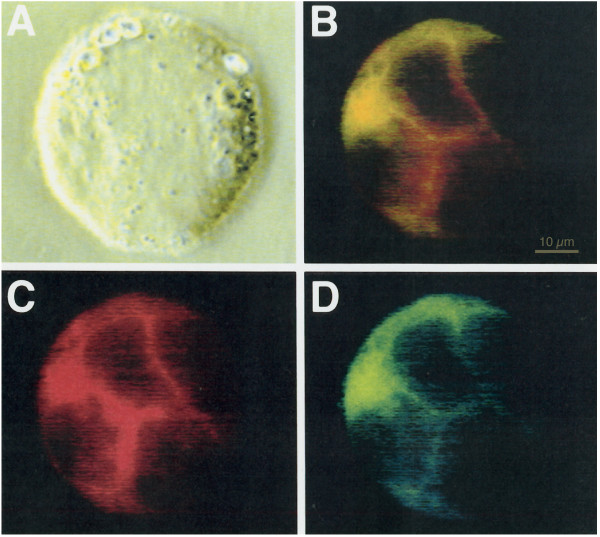
Co-localization CXCR4 and HIV-1 glycoprotein in HIV-1 infected H9 cells. Four days after HIV-1 infection, cells were fixed and permeabilized with saponin. Cells were then labeled with a human monoclonal antibody that interact with SU and then rhodamine-conjugated goat anti-human antibodies (Panel C: red fluorescence) and with 12G5 mAb followed by fluorescein-conjugated goat anti-mouse antibodies (Panel D:green fluorescence). Panel A: phase contrast image. Panel B represents a superposition of green and red fluorescence, with costained regions appearing in yellow. Yellow regions in panel B indicate the colocalization of chemokine receptor CXCR4 and HIV-1 proteins.

Exogenously added HIV SU or SU expressed from recombinant vectors can form a complex with CD4 and chemokine receptor [36,37]. Double labeling was used to determine if an analogous complex of CXCR4 and HIV-1 glycoprotein can be detected in HIV-1 productively infected cells. RH9 T-lymphoblastoid cells were infected with HIV-1_LA1 _at a MOI of 4 or mock-infected. After 4 days PI, the cells were fixed, permeabilized with saponin and incubated with 12G5 CXCR4 MAb followed by a FITC-conjugated second antibody. For staining of HIV-1 glycoproteins, cells were incubated with rhodamine-conjugated antibodies to the HIV-1 proteins and double-fluorescence analysis was performed. A phase contrast micrograph of a multinucleated HIV-1 infected cell is shown in Figure 4A. Figure 4C and Figure 4D represent staining for anti-HIV-1 proteins (red) and anti-CXCR4 (green) MAb, respectively. Superpositions of the two color channels appear in yellow representing the degree of colocalization of CXCR4 and HIV-1 proteins (Fig. 4B). Similar results were observed in nonsyncytial cells expressing HIV-1 proteins. These results suggest that HIV-1 SU and CXCR4 are colocalized in HIV-1 productively-infected RH9 cells.

### Inducible expression of HIV-1 Env down-regulates cell surface CXCR4 expression

**Figure 5:**
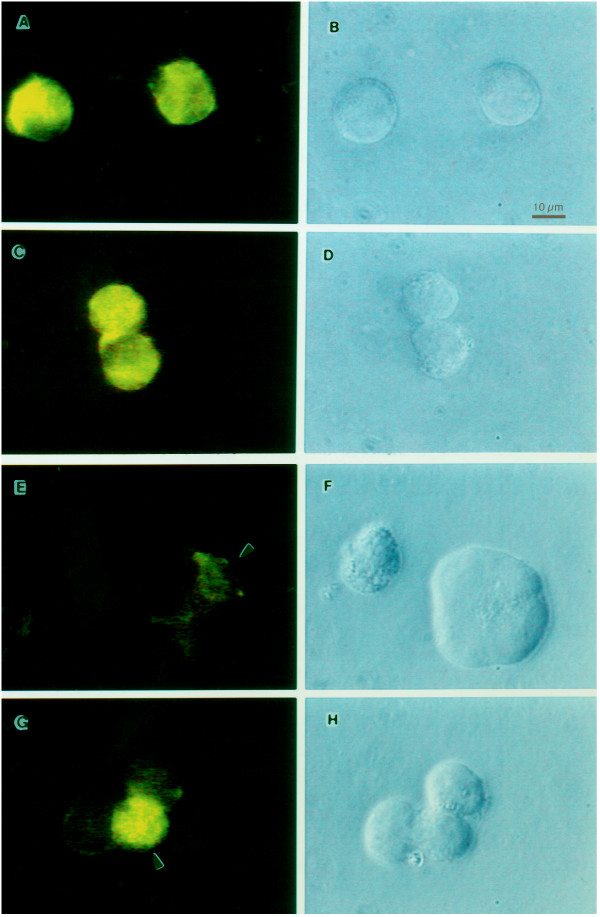
CXCR4 expression is reduced in Jurkat cells after induction of HIV-1 Env expression. After 4 days induction of HIV-1 Env proteins, non-induced and induced cells were fixed and labeled with a mouse MAb to CXCR4 (12G5) and a secondary FITC-conjugated anti-mouse antibodies for observation with a fluorescence microscopy. Panels A and C: CXCR4 staining of non-induced Jurakt cells. Panel E and G: CXCR4 staining of induced Jurkat cells. The fluorescent cell in panel G is representative of a minor population of cells in the induced culture (<5%) with a CXCR4 surface distribution similar to uninduced cells. Panels B, D, F and H show phase contrast images of the same fields of cells shown in left panels.

HIV-1 Env have been suggested to play a role in down-regulation of surface CD4 molecules from the plasma membrane [28,45,46]. The effect of inducible expression of the HIV-1 envelope protein (strain HXB2) on CXCR4 expression was analyzed in CD4+ Jurkat lymphocytes with a well-characterized tetracycline inducible expression system [47,48]. Env expression was monitored by syncitial formation and immunofluorecence staining for Env proteins. In the presence of tetracycline, no fluorescence was observed in Jurkat cells, indicating that Env expression was repressed. When Jurkat cells were cultured in the absence of tetracycline to induce Env expression, >95% of cells stained positive for HIV-1 Env. In the presence of tetracycline, i.e, no Env expression, cells expressed a similar amount of CXCR4 as Jurkat cells without the Env expression plasmid (Fig. 5A–D). In contrast, a decrease in the level of CXCR4 expression was seen in >95% of Jurkat cells expressing Env proteins (Fig 5E–G), indicating that Env expression leads to down-regulation of cell surface CXCR4 expression. There was a strong correlation between a lack of Env expression and expression of CXCR4 in cells of the induced cultures. The distribution of CXCR4 on the minor population of induced Jurkat cells (<5%) with surface CXCR4 was similar to that of uninduced cells (Fig. 2G, H).

## Discussion

Cellular receptors for viruses are often down-regulated from the plasma membrane following productive infection, making infected cells refractory to superinfection by other viruses that use the same receptor for entry [49,27,52]. The decrease in surface expression may be caused in part by the formation of a complex between the viral receptor binding protein and cellular receptors in intracellular compartments. Both HIV-1 and simian immunodeficiency virus down-regulate cell surface expression of CD4, their primary receptor [26,53]. Several mechanisms have been proposed to account for the down-regulation of CD4 following primate lentivirus infection [26,28,54,55]. Internalization of CD4 can occur upon binding of HIV-1 envelope glycoproteins [45,46]. Down-regulation of CD4 may also be mediated by the HIV-1 Nef and Vpu accessory proteins [55]. Nef is expressed early and Vpu late preventing CD4 expression throughout the HIV-1 replication cycle. Nef links CD4 to components of clathrin-dependent trafficking pathways resulting in internalization and delivery of CD4 to lysosomes for degradation [56-59]. Vpu links CD4 to a ubiquitin ligase thereby facilitating degradation of CD4 in the endoplasmic reticulum [60].

Here we demonstrate that during productive acute cytopathic infection of CD4+ T-lymphoblastoid cells by HIV-1 there is an extensive down-regulation of cell surface CXCR4 expression, which correlated with the increase in HIV-1 protein expression. CXCR4 appears to be concentrated in intracellular compartments in H9 cells after HIV-1 infection. Colocalization of both CXCR4 and HIV-1 glycoproteins was detected in HIV-1 infected cells. Epitope masking is unlikely to be responsible for the loss of CXCR4 surface staining since intracellular complexes were readily detected. Down-regulation of the CXCR4 coreceptor during productive infection by CD4-dependent X4 HIV-1 strains was not observed in a previous study by Chenine and coworkers [38]. In contrast to results with the X4 HIV-1 strains they tested, Chenine and coworkers observed a complete loss of CCR5 staining on the surface of cells chronically infected with R5 viruses [38]. Furthermore, it has been shown that CXCR4 is down-regulated by HIV-2 isolates that use CXCR4 as their primary receptor [39]. CXCR4 is also down-regulated in cells infected with CD4-independent X4 HIV-1 isolate m7NDK [40]. However, another CD4-independent HIV-1 isolate, HIV-1/IIIBx, failed to down-regulate CXCR4 on chronically infected cells [61].

There are several plausible explanations for the differences in the results we obtained in the current study with those obtained previously by Chenine *et al.*[38]. As with the two CD4-independent HIV-1 isolates tested that differ in CXCR4 down-regulation [40,61], it is possible that Env of the two X4 strains of HIV-1 we used (LA1, HXB2) differ in their ability to down-modulate CXCR4 from the Env of the X4 viruses (HX10, MN) used by Chenine and coworkers. HIV-1 strain LA1 grows to high titers and the Tet-Off system in Jurkat cells produces significant amounts of HXB2 Env. LA1 is highly cytopathic and significant CPE is observed in the inducible HXB2 Env expression system [48]. In contrast, "little syncytium formation and cell death" was observed in the X4 HIV-1 infected cultures used by Chenine and coworkers [38]. The CD4 independent HIV-2 strain that down-regulates CXCR4 used by Endres et al. (1996) was also highly cytopathic. However, it is unlikely that cytopathic effects are responsible for the decrease in surface CXCR4 by simply selecting for cells in the culture with a low level of CXCR4. CXCR4 is uniformly present on the cells in the RH9 and Jurkat cultures. It is possible that other strains of HIV-1, which grow to lower titers than LA1 or produce less HIV-1 Env than the HXB2 inducible expression system, may have a smaller impact on cell surface CXCR4 for stochastic reasons. The Env of the strains used here may also have a higher affinities for CXCR4 than certain other X4 viruses, allowing direct CXCR4-Env complexing intracellularly. It is also possible that differences in the ability to down-regulate CXCR4 are cell specific. However, we used two different cell lines, RH9 and Jurkat, in the current studies and observed HIV-1 induced CXCR4 down-regulation in both. We also observed a partial down-regulation of CXCR4 in primary human peripheral blood mononuclear cells after infection of HIV-1 (not shown).

Alteration in CXCR4 expression after infection by HIV-1 could result from sequestration of CXCR4 intracellularly or from the direct effects of other HIV-1 proteins on the synthesis of CXCR4 or its transport to the cell surface. Several studies have shown that HIV-1 SU can displace chemokines from their receptors [34,35]. Interactions between SU, CD4, and CXCR4 have also been well established [62,36]. Previous studies demonstrated that treatment with the HIV-1 SU increased colocalization of CD4 with CXCR4 and cocapping of the gp120-CD4-CXCR4 complexes resulted in the cointernalization of a proportion of the gp120-CXCR4 complexes into intracellular vesicles [37]. We did observe down-regulation of surface CXCR4 in an inducible system for Env (and Rev) in which accessory proteins Nef and Vpu are not expressed. However, given other studies suggesting that Nef and Vpu may be able to down-regulate CXCR4 independently of Env, the role these proteins should be considered in future work. HHV-6 and HHV-7 induce down-regulation of CXCR4 [63]. These viruses do not use CXCR4 for cell entry, and induce a markedly decreased level of CXCR4 gene transcription without any significant alteration of the posttranscriptional stability of CXCR4 mRNA. Reduced levels of CXCR4 mRNA transcripts were observed in cells infected with CD4-independent HIV-1 isolate [26]. Furthermore, the modulation of CCR5 expression by the R5 viruses is at the level of transcription [38]. Further experiments will be needed to determine the mechanisms of down-modulation of surface CXCR4 by HIV-1.

## Conclusion

The amount of surface CXCR4 was greatly reduced in T-lymphoblastoid cells infected with HIV-1 strain LA1, but expression of another membrane antigen, CD3, was unaffected. CXCR4 was concentrated in intracellular compartments in RH9 cells after HIV-1 infection. Immunofluorescence microscopy studies showed that CXCR4 and HIV-1 glycoproteins were co-localized in HIV-1 infected cells. Inducible expression of HIV-1 envelope glycoproteins also resulted in down-regulation of CXCR4 from the cell surface. CXCR4 down-regulation may be due in part to intracellular sequestering of HIV glycoprotein/CXCR4 complexes.

## Methods

### Cells and virus

Cells of the RH9 subclone of the CD4+ human T-lymphoblastoid cell line RH9 were the kind gift of Dr. Suraiya Rasheed (University of Southern California), and were maintained in RPMI 1640 supplemented with 10% fetal bovine serum (GIBCO, Long Island, NY), penicillin (100 U/ml) and streptomycin (100 μg/ml). Joseph Sodroski (Harvard University) kindly provided the Env-inducible Jurkat cell line [48].

### Flow cytometry and immunofluorescence microscopy

RH9 T-lymphoblastoid cells were infected with HIV-1_LA1 _at a MOI of 4 or mock-infected. At various times after the addition of virus, cells were fixed in 4% paraformaldehyde for 15 min at room temperature, washed and stained with the mouse MAb 12G5 (10 μg/ml) against human CXCR4 followed by fluorescein isothiocyanate (FITC)-conjugated goat anti-mouse immunoglobulin G (Sigma). In some experiments cells were permeabilized by incubation with 0.05% saponin in PBS for 15 min prior to addition of antibody. CXCR4 monoclonal antibody 12G5 derived by Dr. James Hoxie [39] was obtained through the AIDS Research and Reference Reagent Program, Division of AIDS, NIAID, NIH. Mouse isotype-matched antibodies (Sigma) were used as a negative control for the gating of those cells staining negative for a cell surface marker. Flow cytometry was performed on a Coulter EPICS fluorescence-activated flow cytometer (Coulter Electronics, Hialeah, Fla.). For immunofluorescence microscopy cells were analyzed with a Nikon microscope equipped for epifluorescence. Fluorescent images were acquired with an Olympus microscope, a 100 W UV source, appropriate exciter and blocking filters, captured with a CCD, and processed with Adobe PhotoShop.

## Competing interests

The author(s) declare that they have no competing interests.

## Authors' contributions

BC performed all experiments with substantial help from PJG and AH. RFG, SV and CDF provided guidance, expertise, equipment, and funding for these experiments. All authors have read and approved this manuscript.
